# The impact of severity of patellofemoral osteoarthritis on the patient-reported outcomes of total knee arthroplasty with patellar retention: A retrospective comparative study

**DOI:** 10.5152/j.aott.2021.20070

**Published:** 2021-11-01

**Authors:** Xunkai Feng, Chongyi Fan, Fei Wang

**Affiliations:** Department of Orthopaedic Surgery, Third Hospital of Hebei Medical University, Shijiazhuang, Hebei People’s Republic of China

**Keywords:** Total knee arthroplasty, Patellar retention, Patelloplasty, Patellofemoral osteoarthritis, Patient-reported outcomes

## Abstract

**Objective:**

The aim of this study was to investigate the impact of preoperative patellofemoral osteoarthritis severity on the final patient-reported outcomes in patients with primary osteoarthritis who underwent total knee arthroplasty with patellar retention.

**Methods:**

In this retrospective study, 167 patients (42 males, 125 females; mean age: 67.9 (range, 50-82) years), who underwent total knee arthroplasty with patellar retention due to primary osteoarthritis were included. The preoperative severity of patellofemoral osteoarthritis was classified according to the Iwano classification system. All the patients were then divided into two groups based on the severity of patellofemoral osteoarthritis: Group I, 73 patients (17 males, 56 females; mean age: 68.4 (range, 50-80) years) with mild osteoarthritis (stage 0-I) and group II, 94 patients (25 males, 69 females; mean age: 67.7 (range, 54-82) years) with moderate to severe osteoarthritis (stage II to IV). The mean follow-up was 42.8 (range, 24-59) months in group I and 41.7 (range, 24-63) months in group II. Clinical outcomes were assessed using the Oxford Knee Score, the New Knee Society Score-function score, and the Kujala score preoperatively and at the final follow-up. Also, the Forgotten Joint Score was performed at the final follow-up.

**Results:**

The Oxford Knee Score improved from 22.5 (range, 18-26) preoperatively to 36.5 (range, 30-43) for Group I and from 21.9 (range, 16-25) preoperatively to 35.9 (range, 29-43) for Group II (*P* < 0.001). The Kujala score increased from 51.2 (range, 45-65) preoperatively to 79.3 (range, 71-88) for Group I and from 50.3 (range, 42-60) preoperatively to 80.2 (range, 71-86) for Group II (*P* < 0.001). The New Knee Society Score-function score raised from 60.2 (range, 50-72) preoperatively to 82.2 (range, 72- 90) for Group I and from 59.5 (range, 48-69) preoperatively to 81.4 (range, 73-90) for Group II (*P* < 0.001). The Forgotten Joint Score was 69.9 (range, 63-76) in Group I and 70.2 (range, 62-77) in Group II (*P* = 0.49).

**Conclusion:**

Evidence from this study has shown that the preoperative severity of patellofemoral osteoarthritis has no significant impact on the final patient-reported outcomes of patients with primary osteoarthritis after patellar retention total knee arthroplasty.

**Level of Evidence:**

Level III, Therapeutic Study

## Introduction

Total Knee Arthroplasty (TKA) is considered a successful treatment for end-stage osteoarthritis of the knee. However, the literature remains controversial regarding the management of patella during the primary TKA. Some surgeons have recommended a routine patellar resurfacing based on the low incidence of postoperative anterior knee pain and the reducing risk of re-operation.^1-^^3^ However, others have reported the similar clinical outcomes between patellar resurfacing and Patellar Retention (PR).^4-^^6^

In recent years, orthopedic surgeons have refocused their attention on the PR. However, the preoperative lesion of patellofemoral articular cartilage is the major risk factor for the articular symptom and function after the TKA with PR.^7^ Although some previous studies have shown that the severity of patellofemoral osteoarthritis (PFOA) does not affect the clinical and radiographic results of the TKA with PR,^8-^^11^ these studies evaluated the clinical outcomes primarily from the surgeon-centered perspective. The goal of the operation is to make patients feel satisfied with their functional recovery and pain relief after the TKA. However, patient-based and surgeon-based assessments of surgical outcomes are often inconsistent, especially in terms of pain and function.^12,13^ To the best of our knowledge, there has been no report whether the severity of preoperative PFOA is an influencing factor of the patient-reported outcomes of the primary TKA with PR.

This study was to determine the impact of preoperative severity of PFOA on the postoperative patient-reported outcomes in patients with primary osteoarthritis who underwent primary TKA with PR. Based on the aforementioned background, we hypothesized that the severity of preoperative PFOA did not influence postoperative patient-reported outcomes.

## Materials and Methods

### Patients

**Figure 1.**
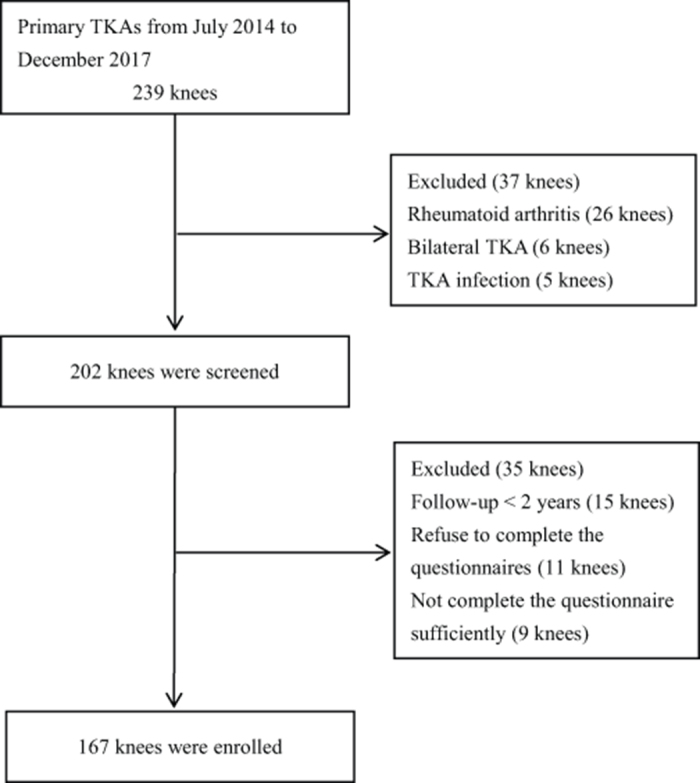
Flowchart of subject screening and enrollment.

We retrospectively reviewed 239 primary TKAs performed by a single senior surgeon from July 2014 to December 2017 at our institute. The inclusion criterion were (1) a diagnosis of primary degenerative osteoarthritis; (2) surgery on just one side; (3) undergoing the TKA with patellar retention using the LINK^®^GEMIN^®^MKⅡ(CR); (4) a minimum of 2-year follow-up; (5) being willing to complete the questionnaires. The exclusion criterion included: (1) bilateral TKA; (2) the follow-up less than 2 years; (3) being unwilling to complete the questionnaires; (4) having a TKA infection; (5) prior high tibial osteotomy, component malalignment, abnormal patella position (patellar instability, patellar alta, etc.), rheumatoid arthritis, and other forms of arthritis. Due to the exclusion criterion, 69 patients (72 knees) were not included in the study. Of these, three patients underwent the bilateral TKA, 15 patients were followed up less than 2 years, 11 patients refused to complete the questionnaires, nine patients did not complete the questionnaire sufficiently, five patients had an infected TKA and 26 patients were diagnosed with rheumatoid arthritis (Figure 1). The presence and severity of PFOA on plain radiographs were evaluated using the Iwano classification system by an experienced radiologist.^14^ Because of the low numbers of the stage II (moderate), the remaining 167 patients (167 knees; 42 males, 125 females; mean age: 67.9 (range: 50-82) years) were divided into two comparison (mild and moderate to severe) groups according to the severity of preoperative PFOA. 73 patients (17 males, 56 females; mean age: 68.4 (range: 50-80) years) with mild osteoarthritis (stage 0−I) were assigned to Group I, and 94 patients (25 males, 69 females; mean age: 67.7 (range: 54−82) years) with moderate-to-severe osteoarthritis (stages II−IV) were assigned to Group II. This study was approved by the Institutional Review Board at our institution, and all patients gave their informed consent.

### Surgical technique

All surgeries were performed by the same senior surgeon with over 10 years of experience in joint surgery, who routinely performed patelloplasty. The surgical schedule comprised a midline skin incision and standard medial parapatellar approach, and the same type of prosthesis (LINK^®^GEMIN^®^MKⅡ(CR)) was implanted in all of the patients. All patients underwent patelloplasty rather than the patellar resurfacing. First, the marginal osteophytes around the patella were removed using the rongeur, then the patellar surface and both side of the patellar facets were reshaped with an electric saw to imitate the normal anatomical morphology of the patella. And the circumferential denervation of the patellar rim was not performed.

### Rehabilitation

Patients were encouraged to ambulate using a walker and requested to do quadriceps strengthening exercises and straight leg raising exercises the first day after surgery. In those patients who underwent surgery in the morning, supervised physical therapy was initiated in the afternoon while in those who had surgery after midday; it was initiated the next day. Two days after surgery, continuous passive motion machines were used for ROM exercises and tolerable weight bearing was commenced in all patients. Perioperative antibiotics for prophylaxis and a standard protocol of postoperative multimodal pain management. In the absence of complications, most patients were discharged 4 to 5 days postoperatively.

### Follow-up

All patients were evaluated preoperatively and followed at 1 month, 3 months, 6 months, and then annually starting at 1 year after surgery. Clinical outcomes were assessed using the Oxford Knee Score (OKS), the New Knee Society Score-Function Score (NKSS-Function Score), and the Kujala Score preoperatively and at the final follow-up. Also, the Forgotten Joint Score (FJS) was performed at the final follow-up.

All patients completed three questionnaires preoperatively, and the preoperative data were collected and saved by our attending surgeon. And the postoperative questionnaires were sent by mail or collected during outpatient’s follow-up. Our attending surgeon supervised the completion of the questionnaires. All patients were contacted by telephone if questionnaires were not sent back within 1 month. All questionnaires were translated and validated.

The OKS was a 12-part questionnaire assessed the pain and function of the knee, each question with five responses and scored from 0 (most severe pain or limited function) to 4 (no pain or functional limitation). At the end of the questionnaire, the final score ranged from 0 to 48, and the higher the score is, the better the outcome is.^15^

The Kujala Score is a validated and 13-part questionnaire specifically designed to assess the anterior knee pain related to different activities. At the end of the questionnaire, the final score ranged from 0 to 100, and the higher the score is, the lighter the pain is.^16^

The NKSS-function score is a questionnaire derived from the New Knee Society Score, which is mainly designed to assess the function related to activities of daily life and athletic sports. At the end of the questionnaire, the final score ranged from 0 to 100 and higher scores represented better function.^17^

The FJS is a 12-item questionnaire was designed to analyze the patient’s awareness of an artificial joint during various daily life activities. In the FJS-12, high scores indicate good outcome, that is, a high degree of “forgetting” the joint.^18^

### Statistical analysis

Before the investigation, the sample size was estimated using the Kujala Score as the primary variable. For a confidence level of 95% (α = 0.05) and power (1 − β) of 80%, a sample size of 49 patients per group was required. Statistical analysis was performed with SPSS for Windows (version 21.0; IBM SPSS Corp.; Armonk, NY, USA). All data were represented by mean and range (minimum to maximum) in our study. The Chi-square test was used for categorical binary data. The Wilcoxon signed ranks test and paired *t*-test were used for comparing the pre- and post-operative data of each group. The Mann–Whitney *U*-test was used for group comparison. All statistical assessments were two-sided; *P* < 0.05 was defined as significant.

## Results

The overall mean follow-up duration was 42.2 (range: 24-63) months. The mean follow-up was 42.8 (range: 24-59) months in group I and 41.7 (range: 24-63) months in group II. The demographics of all patients were presented in Table 1. The demographic data were parallel in the two groups (*P* > 0.05 for age, gender, side, BMI, and duration of follow-up).

### The Oxford Knee Score

In the mild group, the mean OKS was significantly improved from 22.5 (range: 18-26) preoperatively to 36.5 (range: 30-43) postoperatively (*P* < 0.001). In the moderate-to-severe group, the mean OKS was significantly improved from 21.9 (range: 16-25) preoperatively to 35.9 (range: 29-43) postoperatively (*P* < 0.001) (Table 2).

There was no statistically significant difference between the two groups (*P* = 0.21and *P* = 0.27, respectively) (Table 3).

### The Kujala Score

In the mild group, the mean Kujala Score was significantly increased from 51.2 (range: 45-65) preoperatively to 79.3 (range: 71-88) postoperatively (*P* < 0.001). In the moderate-to-severe group, the mean Kujala Score was significantly increased from 50.3 (range: 42-60) preoperatively to 80.2 (range: 71-86) postoperatively (*P* < 0.001) (Table 2).

There was no statistically significant difference between the two groups (*P* = 0.34 and *P* = 0.19, respectively) (Table 3).

### The New Knee Society Score-function score

In the mild group, the mean NKSS-function score was significantly raised from 60.2 (range: 50-72) preoperatively to 82.2 (range: 72-90) postoperatively (*P* < 0.001). In the moderate-to-severe group, the mean NKSS-function score was significantly raised from 59.5 (range: 48-69) preoperatively to 81.4 (range: 73-90) postoperatively (*P* < 0.001) (Table 2). There was no statistically significant difference between the two groups (*P* = 0.61 and *P* = 0.36, respectively) (Table 3).

### The Forgotten Joint Score

In the mild group, the mean FJS was 69.9 (range: 63-76), and in the moderate to severe group, the mean FJS was 70.2 (range: 62-77). There was no statistically significant difference between the two groups (*P* = 0.49) (Table 3).

## Discussion

The most important finding of this study was that no significant difference was found between the two comparison groups in patient-reported outcomes of primary TKA with PR regardless of the severity of preoperative PFOA. And all patients obtained satisfied patient-reported outcomes, even in patients with advanced PFOA.

The advanced PFOA was thought to be an indicator for patellar resurfacing. However, some previous studies had reported that good clinical outcomes were obtained after primary TKA with PR in patients with severe preoperative PFOA. Eshnazarov et al. compared radiological outcomes after TKA with or without patellar resurfacing in patients with severe preoperative PFOA, and they found that the PR achieved similar radiological outcomes than the patellar resurfacing.^8^ This was similar to Seo et al.,^9^ they found that clinical outcomes showed no significant difference between PR and patellar resurfacing in patients with moderate or severe preoperative PFOA. According to Won-Joon et al.,^10^ satisfied clinical outcomes were obtained after primary TKA with PR, even in patients with advanced PFOA, and they stated that the severity of preoperative PFOA did not affect the postoperative clinical outcomes. Further, Hwang et al. suggested that PR with a patelloplasty might be viable as a routine procedure, even in knees with advanced PFOA.^11^ However, the patient-based and surgeon-based assessments of surgical outcomes were often inconsistent.^12,13^

According to Dowsey et al.,^19^ patients with severe preoperative knee osteoarthritis achieved better postoperative patient-reported outcomes. And similar findings had been reported by several authors, with more severe preoperative knee osteoarthritis obtained better patient-reported outcomes than mild preoperative knee osteoarthritis.^20,21^ In contrast, Tilbury et al. recently found that the severity of preoperative knee osteoarthritis had no influence on the patient-reported outcomes after TKA.^22^ In this study, we investigated the association between the severity of preoperative PFOA and the postoperative patient-reported outcomes of primary TKA with PR. Based on our results, there were no significant differences in the postoperative anterior knee pain and knee function between the two comparison groups. The etiology of anterior knee pain was multifactorial, including patellar instability; patellar fracture; component malalignment; inflammation of the tendon, bursa, or synovium around the patella; prosthetic design; and even patient characteristics.^23–25^ The patellofemoral articulation is the most common etiology in the literature. In this study, we excluded the patients with patellar instability and component malalignment; all patients had the same prosthesis and the demographic data were parallel in the two groups. The evidence from this study showed that the preoperative severity of PFOA had no significant impact on the postoperative anterior knee pain of patients with primary osteoarthritis after TKA with PR.

The patelloplasty involved the removal of marginal osteophytes around the patella and the reshaping of the surface of the patella, which replaced the traditional method (remove osteophyte alone), and the circumferential denervation of the patellar rim was not performed in our TKA surgery. According to Sun et al.,^26^ the patelloplasty achieved better clinical outcomes than the traditional method. Similarly, Zupan et al. stated that the patelloplasty was better than traditional method in relieving pain and improving function and quality of life at a mean 35-month follow-up.^27^ A randomized controlled trial compared the clinical outcomes between electrocautery and non-electrocautery. The conclusion of this study was that the electrocautery and non-electrocautery achieved similar clinical outcomes at a mean 3.7-year follow-up.^28^ And a meta-analysis study reported that there was no strong evidence that the efficacy of electrocautery was superior to non-electrocautery in primary TKA.^29^

One advantage of our study was the use of the NKSS-function score. Compared with other Patient-Reported Outcome Measures (PROMs), the NKSS-function score could provide the greatest accommodation of variations between the lifestyles of individual patients. And the NKSS-function score could notice the ceiling effects of existing PROMs in younger, active patients with knee osteoarthritis.^30^ And another advantage of our study was the use of the FJS, which had a lower ceiling effect that enable to distinguish between good and excellent outcomes.^31,32^

Several factors limit the conclusions of this study. First of all, this study is a retrospective study with potential bias in patient selection. Second, the FJS only be applied postoperatively rather than preoperatively. Third, the follow-up time is short, and the results of long-term follow-up are still unknown. Finally, because of the low number of the stage II (moderate), we have to divide all patients into two comparison groups rather than three groups, which could not provide more homogenous patient group and more concrete evidence. Therefore, a clinical trial with long follow-up and big sample size should be performed in the future to validate our findings.

The evidence from this study have shown that the preoperative severity of patellofemoral osteoarthritis has no significant impact on the final patient-reported outcomes of patients with primary osteoarthritis after patellar retention total knee arthroplasty.

**Highlights**

- We investigated the impact of preoperative patellofemoral osteoarthritis severity on the final patient-reported outcomes in patients who underwent total knee arthroplasty with patellar retention.
- All patients used the same type of prosthesis (LINK®GEMIN®MK II [CR]). The patella was treated by patelloplasty, and the circumferential denervation of the patellar rim was not performed.
- Our data showed that the severity of preoperative patellofemoral osteoarthritis did not influence postoperative patient-reported outcomes.

## Figures and Tables

**Table 1.** Patient Demographics

	Group I	Group II	*P*
Age (years)	68.4 (50-80)	67.7 (54-82)	0.52
Gender (F/M)			0.62
Female	56	69	
Male	17	25	
Side (L/R)			0.06
Left	41	39	
Right	32	55	
BMI (kg/m^2^)	26.8 (21-30)	27.2 (23-30)	0.21
Follow-up (months)	42.8 (24-59)	41.7 (24-63)	0.51

BMI, Body Mass Index.

**Table 2.** Comparison of the Pre- and Postoperative the OKS, Kujala Score, and NKSS-Function Score in Both Groups

	Group I			Group II		
	Pre-	Post-	*P*	Pre-	Post-	*P*
OKS	22.5 (18-26)	36.5 (30-43)	*P* < 0.001	21.9 (16-25)	35.9 (29-43)	*P* < 0.001
Kujala Score	51.2 (45-65)	79.3 (71-88)	*P* < 0.001	50.3 (42-60)	80.2 (71-86)	*P* < 0.001
NKSS-function score	60.2 (50-72)	82.2 (72-90)	*P* < 0.001	59.5 (48-69)	81.4 (73-90)	*P* < 0.001

NKSS-Function Score, New Knee Society Score-Function Score; OKS, Oxford Knee Score.

**Table 3.** Comparison of Patient-reported Outcomes between the Two Groups before and after Surgery

		Group I	Group II	*P*
Preoperative	OKS	22.5 (18-26)	21.9 (16-25)	0.21
	Kujala Score	51.2 (45-65)	50.3 (42-60)	0.34
	NKSS-function score	60.2 (50-72)	59.5 (48-69)	0.61
Postoperative	OKS	36.5 (30-43)	35.9 (29-43)	0.27
	Kujala Score	79.3 (71-88)	80.2 (71-86)	0.19
	NKSS-function score	82.2 (72-90)	81.4 (73-90)	0.36
	FJS	69.9 (63-76)	69.9 (63-76)	0.49

FJS, Forgotten Joint Score; NKSS-Function Score, New Knee Society Score-Function Score; OKS, Oxford Knee Score.
